# Nodakenin alleviated obstructive nephropathy through blunting Snail1 induced fibrosis

**DOI:** 10.1111/jcmm.15539

**Published:** 2020-07-22

**Authors:** Jianchun Li, Lu Wang, Ruizhi Tan, Sha zhao, Xia Zhong, Li Wang

**Affiliations:** ^1^ Research Center of Intergated Traditional Chinese and Western Medicine Affiliated Traditional Medicine Hospital Southwest Medical University Luzhou Sichuan China; ^2^ Division of Nephrology The Affiliated Baiyun hospital of Guizhou Medical University Guiyang China; ^3^ Department of Intensive Medicine Affiliated Traditional Medicine Hospital Southwest Medical University Luzhou Sichuan China

**Keywords:** inflammation, Nodakenin, renal fibrosis, Snail1

## Abstract

Tubulointerstitial fibrosis plays an important role in end‐stage renal failure, and there are only limited therapeutic options available to preserve organ function. In the present study, we identified that nodakenin, a coumarin isolated from the roots of *Angelicae gigas*, functions effectively against unilateral ureteral obstruction (UUO)‐induced fibrosis via down‐regulating Snail1 expression. We established UUO‐induced renal fibrosis in mice and then administered with nodakenin orally ata a dose of 1 and 10 mg/kg. The *in‐vivo* results indicated that nodakenin protected obstructive nephropathy through its anti‐inflammatory and anti‐fibrotic properties. Nodakenin prevented the infiltration of inflammatory cells, alleviated the levels of pro‐inflammatory cytokines, reduced the polarization of macrophages and down‐regulating the aberrant deposition of extracellular matrix at the site of injury. Of note, nodakenin dramatically impeded Smad3, NF‐κB p65 phosphorylation and Snail1 expression. In line with *in vivo* studies, nodakenin suppressed the expression of Snail1, Smad3 phosphorylation and fibrogenesis in TGF‐β1‐treated renal epithelial cells *in‐vitro*. Furthermore, we found that the effect of nodaknin against fibrosis was reversed in Snail1 overexpressing cells, whereas nodakenin could not further reduce expression of fibrogenesis in Snail1 silenced cells, suggesting that nodaknein may function through a Snail1‐dependent manner. Collectively, this study reveal a critical role of nodakenin in the cure of renal fibrosis.

## 1 INTRODUCTION

Chronic kidney disease (CKD) has been perceived as an essential public health issue around the world, and its treatment has remained to be an urgent and arduous task.^1^, ^2^ Tubulointerstitial fibrosis, which is considered to be the final common pathway in various types of CKDs, is affected by multiple factors such as inflammatory cell infiltration, activated myofibroblast accumulation and stress molecules, etc. Unfortunately, these pathologies eventually develop into end‐stage renal disease for which there are only limited therapies available. Hence, it would be beneficial to develop more effective therapeutic drugs for renal fibrosis.

It is a widely held view that TGF‐β/Smad signalling plays a critical role in renal fibrosis.^3^ Transforming growth factor (TGF‐β)1 functions in various cell types including renal tubular epithelial cells (TECs) and initiates action via direct (Smad) and indirect (non‐Smad) pathways to cause renal fibrosis.^4^, ^5^ Direct blockade of TGF‐β1 signal transduction with anti‐TGF‐β antibody seems to be an effective strategy. However, researchers recently have proved that administration of TGF‐β1 neutralizing monoclonal antibody failed to alleviate the progression of diabetic nephropathy.^6^ In view of this, treatment of renal fibrosis should focus specifically on the downstream molecules related to fibrosis, rather than blocking the general effect of TGF‐β1. As the critical cellular mediator of TGF‐β1, SMAD family member 3 (Smad3) plays a vital role in renal fibrosis by regulating the transcription of fibrogenic genes within α‐smooth muscle actin (α‐SMA).^7^ Snail1 (encoding snail family zinc finger 1, known as Snail1), activated by Smad3/4 and cooperation of Smad3/4, has been reported to work as a transcription factor to control the expression of junction components like E‐cadherin, claudins and so on. Collectively, inhibition of Smad3 or Snail1 may be a potential therapeutic target for renal fibrosis.

In recent years, drugs isolated from the medicinal herbal have drawn increasing attractions from the scientific researchers as an alternative therapy for the prevention and treatment of various renal disease.^8^, ^9^, ^10^, ^11^ Nodakenin, a coumarin derived from the roots of *Angelicae gigas*, has been reported to treat against multiple diseases including alleviating inflammation to treat against lethal endotoxin shock and allergic inflammation, enhancing cognitive function and adult hippocampal neurogenesis and so on.^12^, ^13^, ^14^ However, little reports have been suggested the effect of nodakenin against renal fibrosis.

Considering the opposite roles on renal fibrosis between nodakenin and Snail1, we hypothesized that nodakenin protect against renal fibrosis by manipulating Snail1 expression. Hence, the goals of this study are to reveal the renoprotective effect of nodakenin on UUO and whether by targeting Snail1. In the current study, we have disclosed that the effects of nodakenin on the inflammation and fibrosis *in vitro* and *in vivo*. Furthermore, we also examined the underlying mechanisms by lentivirus‐mediated up‐regulation or siRNA‐induced down‐regulation of Snail1.

## 2 MATERIALS AND METHODS

### 2.1 UUO‐induced renal fibrosis model and nodakenin treatment

The UUO‐induced renal fibrosis model was constructed in the male BALB/C mice (6 to 8‐week‐old, 20‐25 g) as described previously.^15^ After anaesthetizing with pentobarbital, the left ureter was obstructed by two‐point ligations and snipping the midline of the ureter. The mice were randomly divided into the following groups (n = 6 per group): (a) sham group; (b) UUO with nodakenin at a dose of 1 mg/kg, 10 mg/kg or vehicle solvent (10% tween‐80) (in which mice received 7 consecutive days of nodakenin/vehicle solvent). The next day after surgery, the mice were received with drug treatment by daily gavage and sacrificed at day 7 after treatment. For western blotting and Quantitative‐PCR, mice kidneys were excised and stored in liquid nitrogen before usage. All experimental manipulations were approved by the Ethics Committee for Animal Experiments of Southwest Medical University.

### 2.2 Cell culture, lentivirus infection, siRNA transfection and MTT assay

Rat renal tubular epithelial cell line (NRK‐52E) was obtained from the Cell Bank of the Chinese Academy of Sciences (Shanghai, China). NRK‐52E cells were cultured in DMEM (high glucose) supplemented with 5% (v/v) FBS (Gibco, USA), 100 U/ml penicillin/streptomycin (Life Technologies, USA). For in vitro cellular assay, DMSO was used as a solvent to dissolve nodakenin, and the final concentration of DMSO in the medium was under 0.1%.

The gene of the rat snail1 coding sequence region was synthesized by Sangon (Shanghai, China) and cloned into pLVX‐IRES‐EGFP plasmid. To make lentiviruses, DNA vectors were transfected into human 293T cells. Next, medium supernatant was harvested, centrifuged at 72 000 *g* for 2 hours (Optima XPN‐100, Beckman, USA) and the sediments were resuspended in fresh medium, stored at −80 °C before usage. Finally, lentiviruses were used to infect NRK‐52E cells and 3 days later, 1.5 μg/ml puromycin (Solarbio, China) was added to the medium for 7 days to obtain a stable snail1‐overexpression cell lines. Cells within 3–10 passages were used for further research.

Snail1 siRNA was obtained from genepharme (Shanghai, China). The transfection was conducted according to the manufacturer’s instructions (Lipofectamin RNAiMAX reagent (Invitrogen, USA)). The sequence was as follows: Negative siRNA: sense 5′‐UUCUCCGAACGUGUCACGUTT‐3′; Snail1 siRNA1: sense 5′‐GCGCUCUGAAGAUGCACAUUUTT‐3′; Snail2 siRNA1: sense 5′‐AGACCCACUCGGAUGUGAATT‐3′; Snail3 siRNA1: sense 5′‐ GGAUGUGAAGAGAUACCAGTT‐3′;

MTT assay was conducted as described previously.^16^ In brief, 2 × 10^3^ cells were seed in 96‐well plates and incubated overnight before treatment. Thereafter, cells were received with various concentrations of nodakenin (1‐80 μmol/L) for 48 hours.

Then, the supernatant was discarded and 100 μL of MTT solvent (0.5 mg/mL) was added for incubation. After indicated time (4 hours), the supernatant was again discarded and 150 μL of DMSO was added to dissolve the deposits for 15 minutes. Finally, The absorbance was obtained by a microplate reader (VT, Biotek, USA).

### 2.3 Histological analysis and immunofluorescence staining

The mice kidneys were fixed with paraformaldehyde, embedded in paraffin and sectioned at 4 μm for further analysis. HE staining and Periodic acid‐Schiff staining (PAS) was firstly conducted to evaluate the tubular injury level. In brief, the percentage of cortical tubular injury with 0 to 4 grading scale: 0, normal; 1, less than 25%; 2, 25% to 50%; 3, 50% to 75%; and 4, >75%.^17^ Next, we performed Masson’s trichrome staining and Sirius staining to assess the degree of fibrosis according to the manufacturer’s instructions (Nanjing Jiancheng, China). Immunohistochemistry was performed with a microwave‐based antigen retrieval technique.^18^ After incubating with the antibody against α‐SMA (1: 100, Boster, China), the slides were exposed with Biotin‐Streptavidin HRP‐based SPlink Detection Kits (ZSGB‐Bio, China) and counterstained with haematoxylin. The sections were then photographed with Virtual Slide Microscope (VS120, Olympus, Japan).

For immunofluorescence staining, the kidneys were firstly fixed in 4% paraformaldehyde at 4 °C overnight, followed by dehydrating in 10% to 30% sucrose and embedding in OCT. 5 μm sections were prepared by using a Leica cryostat. The sections were then blocked with 3% goat serum, incubated with anti‐F4/80 antibodies (Santa Cruz, USA), followed by incubation with FITC‐conjugated secondary antibodies. After rinsing with PBS for 3 times, the sections were immediately photographed with confocal microscopy (A1R‐PLUS, Nikon, Japan).

### 2.4 Western blot

Total proteins were extracted from cells or mice kidneys using RIPA lysis buffer (Beyotime, China) and protein concentrations were quantified using the bicinchoninic acid (BCA) protein kit (Beyotime, China). Approximately 30 μg of protein samples were isolated with sodium dodecyl sulphate‐polyacrylamide gel (SDS‐PAGE) and transferred to a PVDF membrane (Millipore, USA). The transferred membrane was incubated overnight at 4 ℃ with corresponding primary antibodies (anti‐α‐SMA antibody, Boster, China; anti‐IL‐1β antibody, Santa Cruz, USA; anti‐Snail1/KIM‐1/iNOS/TNF‐α/phosphorylation NF‐κB p65/NF‐κB p65/Smad3 phosphorylation Smad3 antibody, CST, USA; anti‐Fibronectin/Collagen I antibody, Abcam, USA), followed by HRP‐conjugated secondary antibodies for 1 hour at room temperature. Signals were detected with ChemiDoc^TM^ (Bio‐Rad, USA) and quantified using Gel‐Pro analyser (Media Cybernetics, USA).

### 2.5 Flow cytometry for detection of macrophage polarization and the expression levels of E‐cadherin

For the detection of macrophage polarization, mice kidneys were harvested, digested with Blendzyme 4 (Roche, USA) and grinded into cell suspension as previously report.^19^ In order to detect the expression levels of E‐cadherin, NRK‐52E cells were digested with 0.25% trypsin. After fixation with IC fixation buffer (Ebioscience), cells were stained with corresponding antibodies (anti‐iNOS antibody, CST, USA; anti‐F4/80 antibody, CST, USA; Anti‐ E‐cadherin antibody, BD, USA) at 4˚C overnight. Following rinsing with pre‐cold PBS for 3 times, the cells were then incubated with corresponding fluorescence‐conjugated antibodies and detected with flow cytometer (BD, USA). The data was finally analysed with FlowJo software (X 10.0.7).

### 2.6 RNA extraction and quantitative PCR

After treatment, cells were obtained and total RNA was isolated with RNA extraction kit (Tiangen, China) according to the manufacturer’s instructions. Next, complementary DNA (cDNA) was obtained by using the Reverse Transcription Kit (Promega, China). Then, quantitative PCR was conducted with a LightCycler 480 instrument (Roche, Germany). The mRNA levels of iNOS, IL‐1β and α‐SMA etc. were measured. The comparative cycle threshold (ΔΔCt) method was used to analyse the results. The PCR primers were listed in Table 1.

**TABLE 1:** The sequence of primers

Gene	Sequence
α‐SMA	
α‐SMA‐F	GTCCCAGACATCAGGGAGTAA
α‐SMA‐R	TCGGATACTTCAGCGTCAGGA
Fibronectin	
Fibronectin‐F	ATGTGGACCCCTCCTGATAGT
Fibronectin‐R	GCCCAGTGATTTCAGCAAAGG
Collagen I	
Collagen I‐F	GCTCCTCTTAGGGGCCACT
Collagen I‐R	CCACGTCTCACCATTGGGG
TNF‐α	
TNF‐α‐F	CATCTTCTCAAAATTCGAGTGACAA
TNF‐α‐R	TGGGAGTAGACAAGGTACAACCC
IL‐1β	
IL‐1β‐F	TTCAGGCAGGCAGTATCACTC
IL‐1β‐R	GAAGGTCCACGGGAAAGACAC
iNOS	
iNOS‐F	CAGCTGGGTCGTACAAAC
iNOS‐R	CATTGGAAGTGAAGCGTTT
GAPDH	
GAPDH‐F	ACAGCAACAGGGTGGTGGAC
GAPDH‐R	TTTGAGGGTGCAGCGAACTT
Snail1	
Snail1‐F	GAAGCCCAACTATAGCGAGC
Snail1‐R	AGAGTCCCAGATGAGGGTG

### 2.7 Statistical analysis

All the data were expressed as the mean ± standard deviation (SD). Statistical analyses were performed with student’s t‐test or one‐way ANOVA with SPSS 20.0 software. *P* < 0.05 was considered to be statistically different.

## 3 RESULTS

### 3.1 Nodakenin treatment alleviates renal fibrosis

**FIGURE 1:**
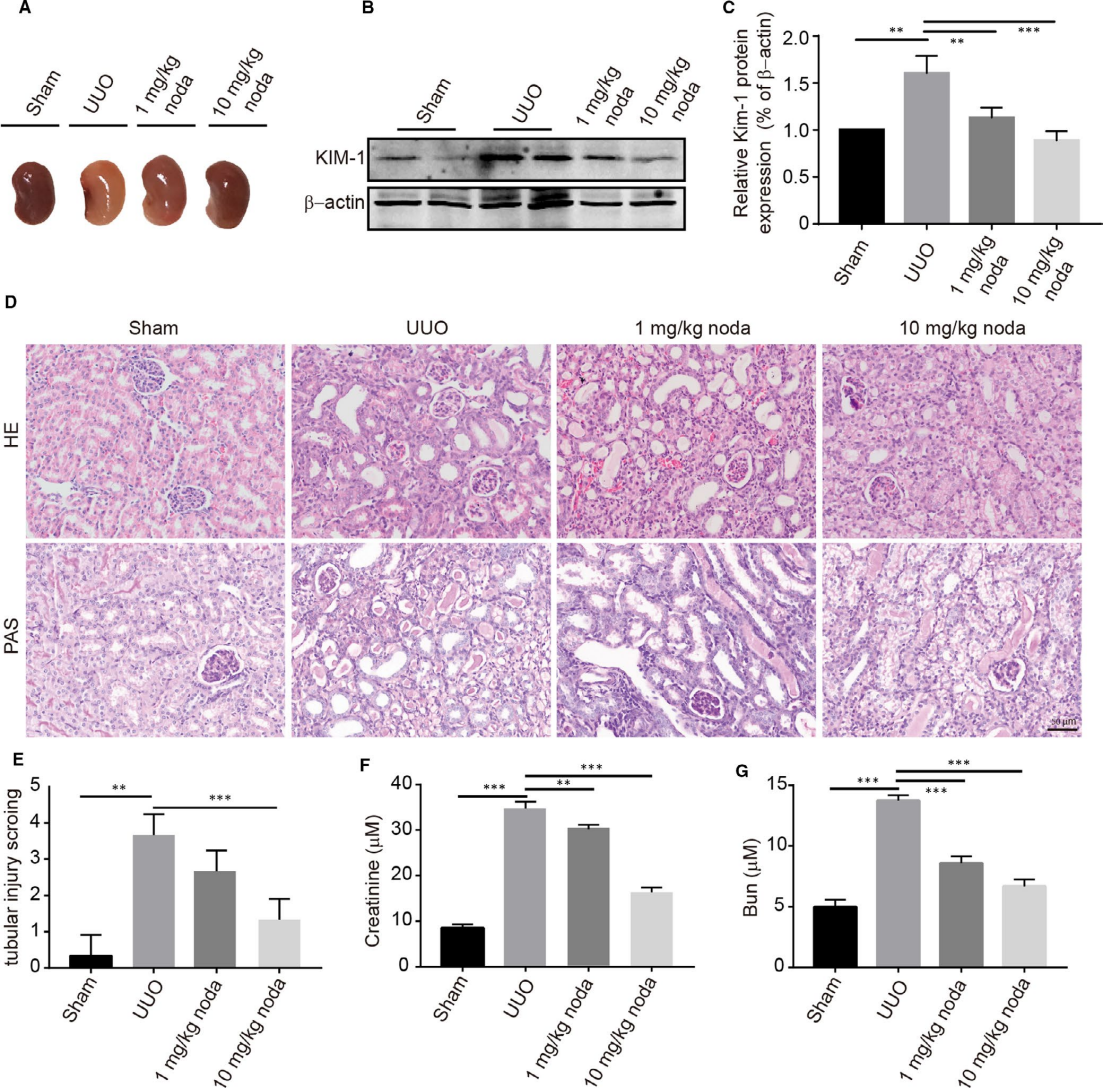
Nodakenin treatment ameliorated the pathologic injuries in UUO model. A, Representative images of kidneys collected from various groups. B, proteins obtained from the mice kidneys and western blot was employed to analyse the expression of KIM‐1. C, Quantification of protein expression by scanning densitometry in (B). D, Haematoxylin and eosin and PAS staining of renal tissues. E, quantification of tubular injury. F, Effects of nodakenin on serum creatinine. G, Effects of nodakenin on blood urea nitrogen. Data represents the mean ± SD for groups of 3 mice. **P < 0.01, ***P < 0.001 compared with the indicated group

To explore whether nodakenin could affect the process of pathologic changes, a UUO‐induced mouse renal fibrosis model was constructed. As shown in Figure 1A, the obstructed kidney in solvent‐treated mice displayed a pale colour compared with the sham group, suggesting a weaker blood supply. Whereas nodakenin treatment significantly reversed the impact. Meanwhile, we also investigated the expression of KIM‐1, a biomarker of proximal tubular injury by western blot. As shown in Figure 1B,C, nodakenin treatment dramatically blunted with UUO‐induced increase in a dose‐dependent manner. In addition, we also measured the mice's kidney pathologic changes. Haematoxylin‐eosin (HE) staining and the periodic acid Schiff (PAS) staining revealed that nodakenin administration dramatically decreased structural damage such as tubular dilation and atrophy, expansion of interstitium and tubule brush border disruption (Figure 1D,E). Moreover, we also investigated the blood biochemical indexes in each group. In line with the outcomes of the kidney pathologic changes, the levels of creatinine and bun after nodakenin treatment restored the aberrant alterations in a dose‐dependent manner (Figure 1F,G). Collectively, these results suggested that nodakenin treatment could effectively alleviate UUO‐induced the pathologic changes.

**FIGURE 2:**
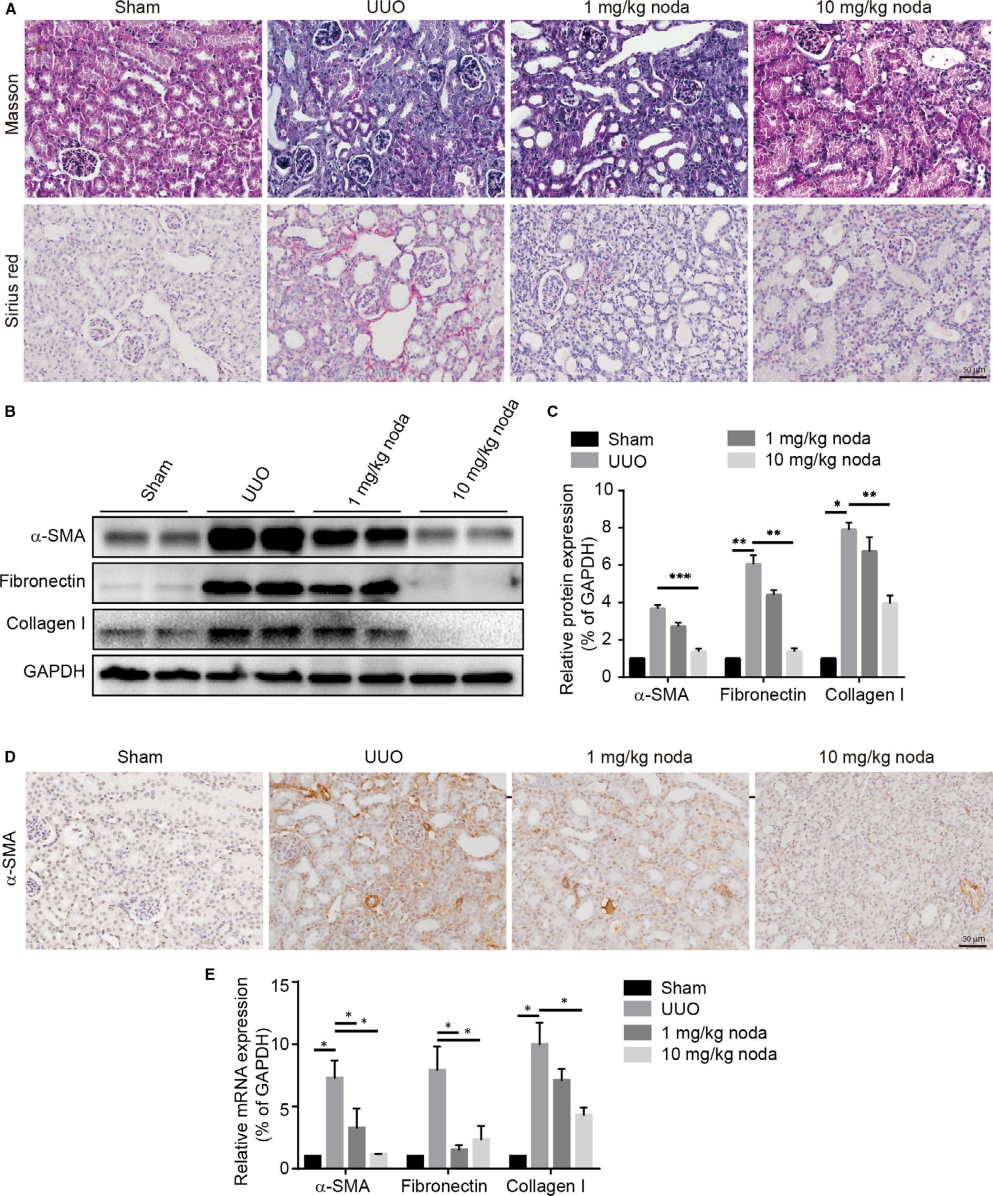
Nodakenin attenuated the degree of fibrosis. A, Masson's trichrome staining and Sirius red staining. B, The protein expression levels of α‐SMA, fibronectin, collagen I in renal lysates by western blot. C, Quantification of protein expression by scanning densitometry in (B). D, Immunohistochemical staining of α‐SMA. E, Quantification of mRNA levels of α‐SMA, fibronectin, collagen I by quantitative PCR. Data represent the mean ± *SD* for groups of 6 mice. *P < 0.05, **P < 0.01, ***P < 0.001 compared with the indicated group

Next, we determined whether nodakenin treatment could inhibit kidney fibrosis. Firstly, Masson trichrome staining and Sirius red staining was performed to investigate the degree of fibrosis in various groups. As presented in Figure 2A, the results revealed that abundant extracellular matrix deposition in UUO kidneys. Nevertheless, nodakenin treatment markedly decreased the intensity of Masson staining and Sirius red staining. Meanwhile, the expression levels of extracellular matrix protein fibronectin, collagen I were also detected by western blot assay. In accordance with expectations, the levels of crucial proteins showed similar effects upon nodakenin treatment (Figure 2B,C). As evidenced by the previous research, aberrantly activated fibroblasts differentiated into myofibroblasts is the leading cause of the excessive extracellular matrix deposition in UUO‐induced kidney fibrosis.^20^ As presented in Figure 2B‐D, UUO kidneys displayed remarkable α‐smooth muscle actin (α‐SMA, the marker of myofibroblast) positive myofibroblasts detected by immunohistochemical staining and immunoblotting. Moreover, we also detected the mRNA expression of α‐SMA, fibronectin, collagen I by quantitative PCR. The results revealed that nodakenin treatment alleviated the mRNA expression levels in a dose‐dependent manner (data presented in Figure 2E). Overall, these results indicated that nodakenin obviously improved renal fibrosis at least partially ascribed to suppress the activation of myofibroblast cells.

### 3.2 Administration of nodakenin attenuates UUO‐induced inflammatory responses in mice

**FIGURE 3:**
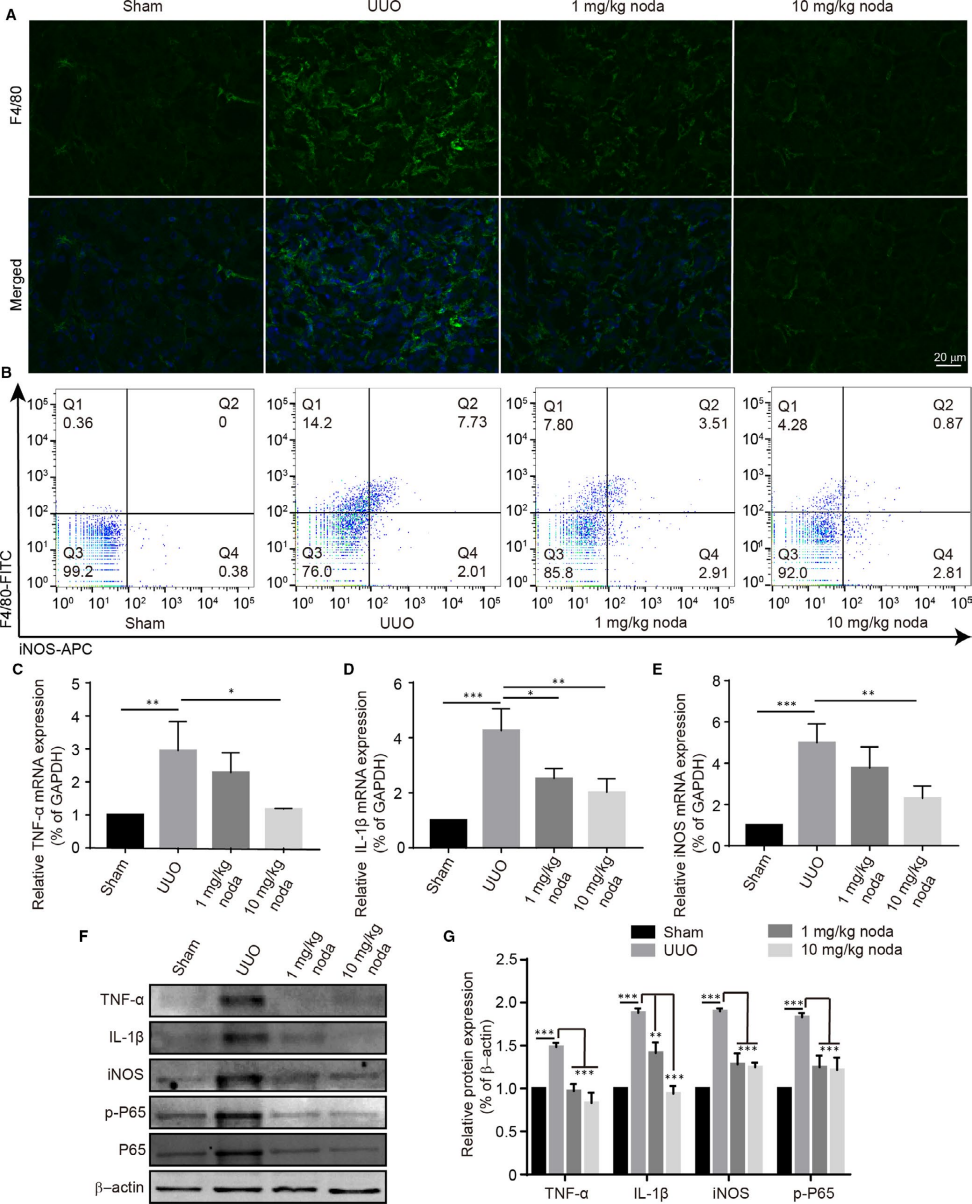
Nodakenin alleviated the renal inflammation in UUO model. A, Immunofluorescent staining for F4/80 in the mice kidneys. B, The expression of F4/80 and iNOS in renal single‐cell suspension was analysed with flow cytometry. C‐E, mRNA levels of TNF‐α, IL‐1β, iNOS by quantitative PCR. F, The indicated protein levels were investigated by western blot. G, Quantification of protein expression by scanning densitometry in (F). Data represent the mean ± *SD* for groups of 6 mice. *P < 0.05, **P < 0.01, ***P < 0.001 compared with the indicated group

Inflammation responses have the critical effect on renal fibrosis as a priming factor, and the macrophage infiltration in renal fibrosis has a dominant role in the production of chemokines such as TNF‐α, IL‐1β, iNOS.^21^, ^22^ To investigate whether nodakenin displayed an anti‐inflammation effect, the macrophage infiltration, polarization and pro‐inflammatory cytokine expression in the mice kidneys were detected by advantage of flow cytometry, immunofluorescence assay, quantitative PCR and western blot. As seen in Figure 3A, the results of flow cytometry indicated that nodakenin treatment dramatically decreased the infiltration of the macrophage. Besides, flow cytometry analysis suggested that nodakenin treatment also reduced M1 macrophage polarization, which is characterized by the production of a range of pro‐inflammatory molecules such as iNOS. In line with flow cytometry, immunofluorescence assay also revealed that nodakenin treatment significantly decreased the macrophage infiltration compared with UUO group by *in situ* immunofluorescence (data presented in Figure 3B). Next, we investigated pro‐inflammatory cytokine expression by quantitative PCR and western blot. As expected, nodakenin treatment reduced the levels of pro‐inflammatory cytokines in a dose‐dependent manner (Figure 3C‐G). Notably, nodakenin treatment reversed the aberrant increase of NF‐κB p65, which lays an upstream of chemokines (Figure 3F,G). Taken together, these results demonstrated that nodakenin could alleviate UUO‐induced inflammatory responses in mice.

### 3.3 Nodakenin inhibits fibrosis in vitro

**FIGURE 4:**
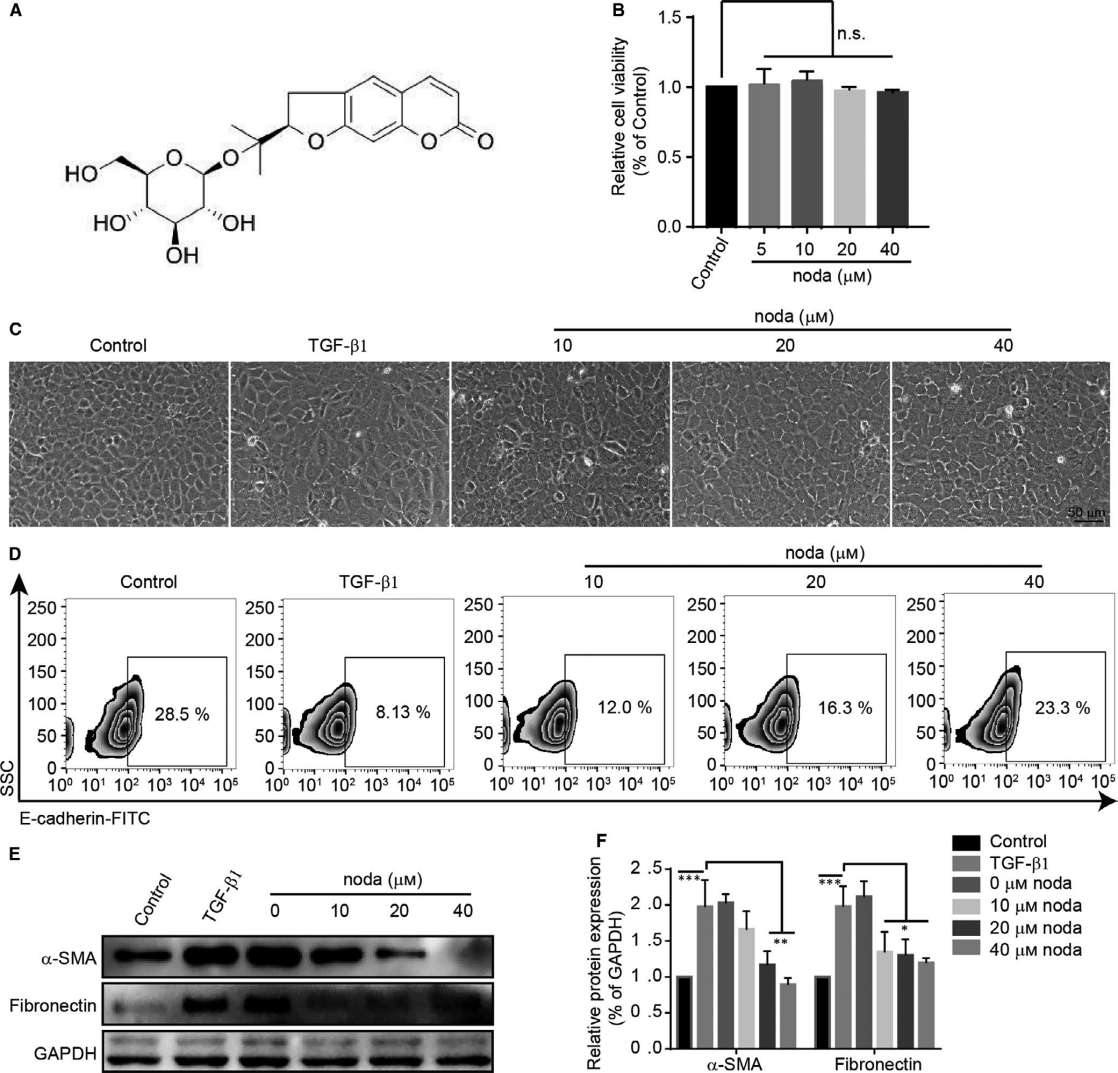
Nodakenin inhibits fibrosis in NRK‐52E cells. A, the structure of nodakenin. B, The cells were received with nodakenin (1‐80 μM) for 48 h and the cytotoxicity was assessed by MTT viability assay. C, The morphology changes of NRK‐52E cells after treating with TGF‐β1 and indicated concentrations of nodakenin or DMSO for 48 h. D, The expression of E‐cadherin in cells was analysed with flow cytometry. E, The indicated protein levels were investigated by western blot. F, Quantification of protein expression by scanning densitometry in (E). Data represent the mean ± SD for at least three independent experiments. n.s. means no significant difference compared with the indicated group, **P < 0.01, **P < 0.01, ***P < 0.001 compared with the indicated group

Tubular epithelial cells (TECs) play a crucial role during the development of renal fibrosis progression.^23^ The molecular structure of nodakenin was presented in Figure 4A. Considering the potential cytotoxicity, we first evaluated the cytotoxicity of nodakenin by MTT assay. As shown in Figure 4B, no obvious injury was observed under 40 µM nodakenin for 48 hours. We then investigated the effects of nodakenin in TGF‐β1 induced fibrosis in rat tubular epithelial cells. As shown in Figure 4C, the results indicated that after stimulation with 6 ng/μL TGF‐β1, the morphology of the cells turned from the typical cobblestone‐like morphology into a long spindle fibroblast‐like morphology. Whereas the administration of nodakenin partially reversed the above change. Then, we determined the expression of E‐cadherin. The flow cytometry analysis (Figure 4D) indicated that nodakenin treatment significantly rescued the loss of E‐cadherin. Moreover, western blotting (Figure 4E,F) results indicated that nodakenin treatment markedly attenuated the TGF‐β1–induced fibrosis markers α‐SMA, fibronectin. Collectively, these results suggested that nodakenin treatment inhibited TGF‐β1–induced cell fibrosis in NRK‐52E cells.

### 3.4 Nodakenin inhibits Smad3 phosphorylation and Snail1 expression *in vitro* and *in vivo*

**FIGURE 5:**
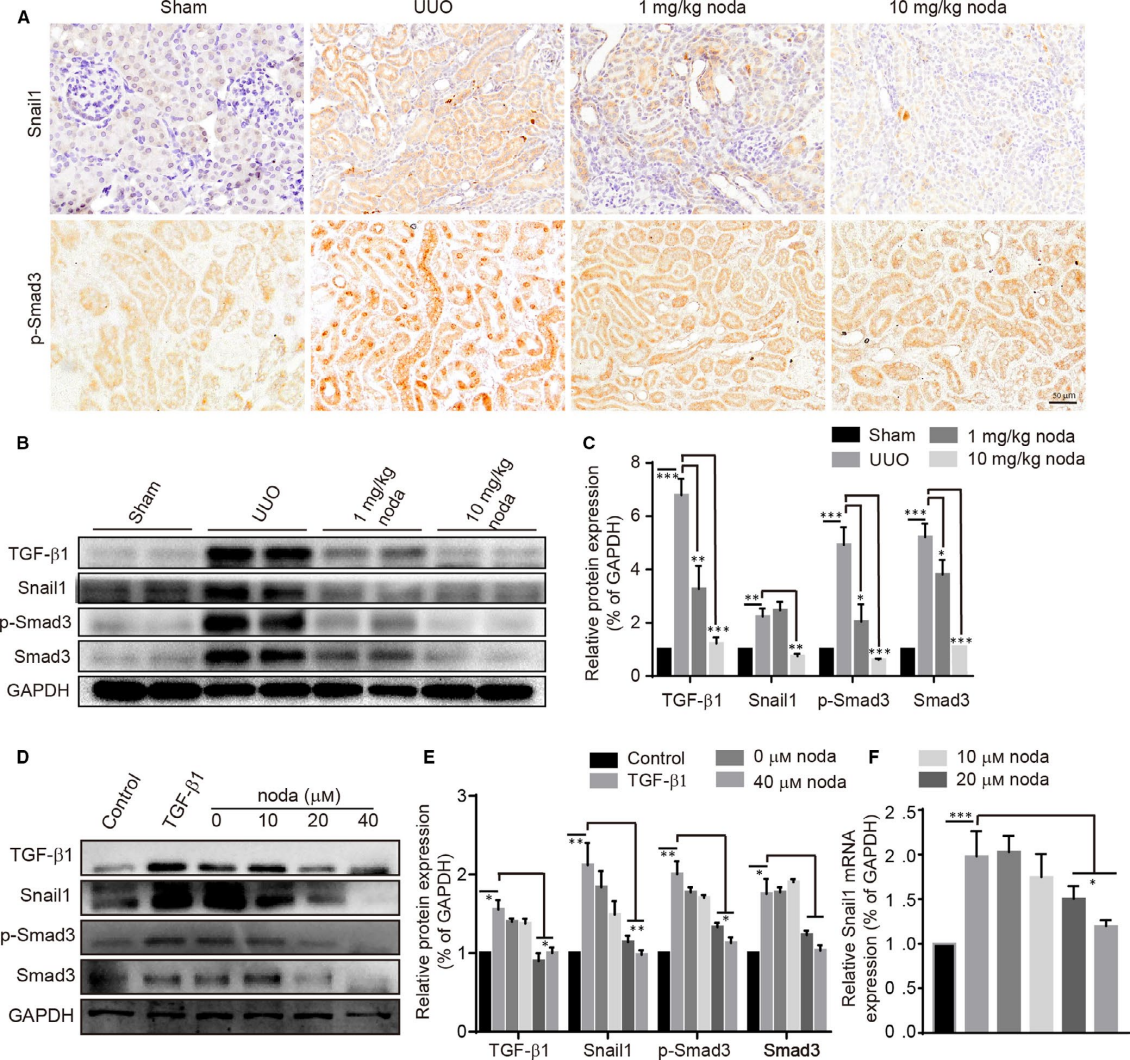
Nodakenin inhibits the Smad3 and Snail1 expression in vitro and in vivo. A, Immunohistochemical staining of phosphorylated smad3 and Snail1. B, proteins obtained from the mice kidneys and western blot was employed to analyse the expression. C, Quantification of protein expression by scanning densitometry in (B). D, The indicated protein levels were investigated by western blot. (E) Quantification of protein expression by scanning densitometry in (D). F, The mRNA level of Snail1. Data represent the mean ± SD for groups of 3 mice/at least three independent experiments. *P < 0.05, **P < 0.01, ***P < 0.001 compared with the indicated group

Based on the above results, we have preliminarily concluded that effect of nodakenin on renal fibrosis, but the specific mechanism remains to be clarified. Hence, we investigated the potential critical pathway in vitro and in vivo. We first evaluated the expression of phosphorylated smad3 and snail1 by immunohistochemical staining. As shown in Figure 5A, a dramatic increase of Snail1 and phosphorylated smad3 was observed in renal tubules of UUO model. Meanwhile, we also observed an obvious translocation of phosphorylated smad3 from the cytoplasm to nuclei. Whereas, nodakenin treatment reversed the above phenomenon. We also evaluated the indicated protein levels in vitro and in vivo by western blot. Consistent with immunohistochemical staining, the results revealed that snail1, TGF‐β1 and phosphorylated smad3 were significantly blunted both in cell‐culture samples and tissue samples (details listed in Figure 5B‐E).

### 3.5 Nodakenin alleviates the fibrosis via a Snail1‐dependent mechanism in renal tubules

**FIGURE 6:**
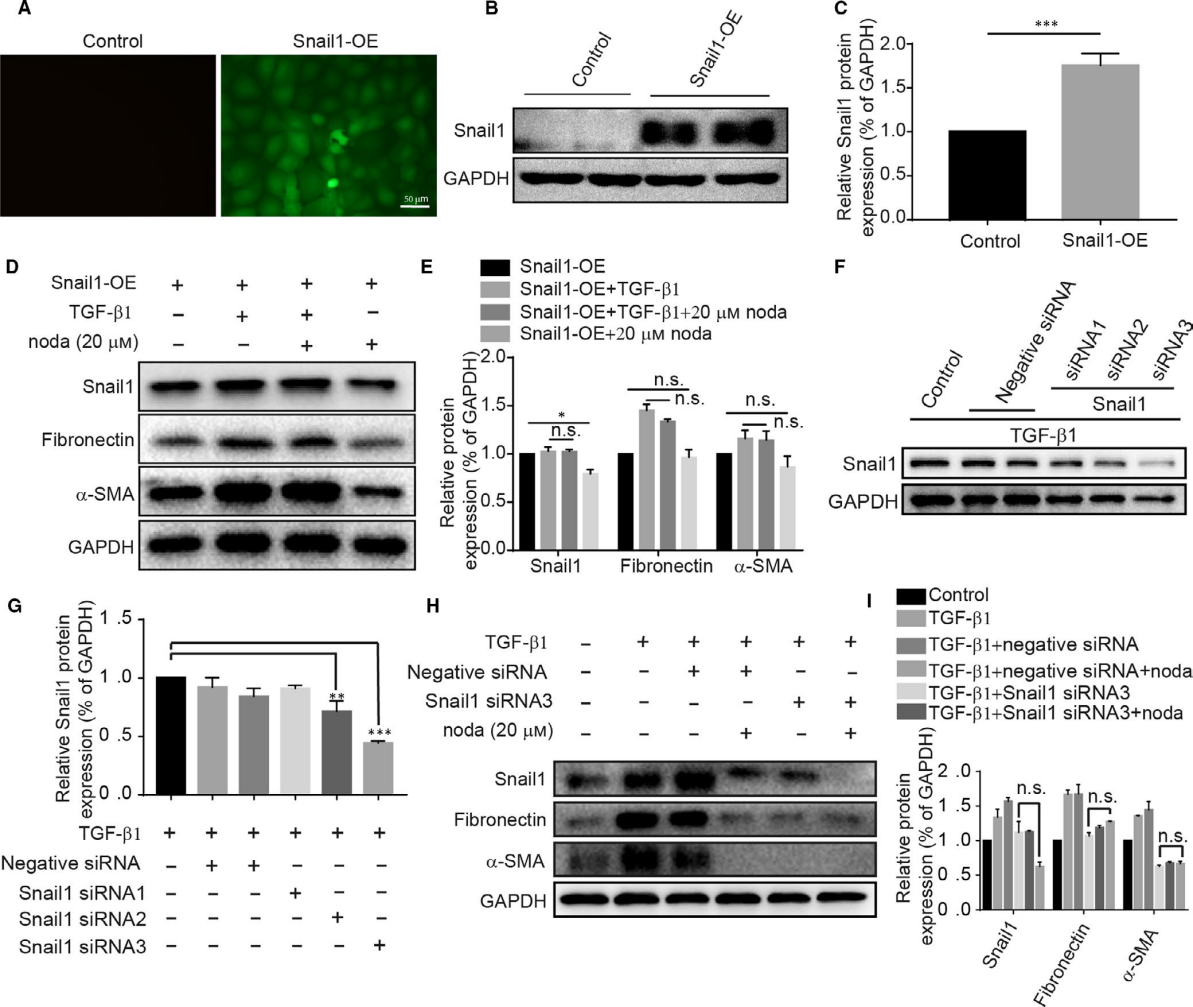
Nodakenin alleviates the fibrosis via a Snail1‐dependent mechanism in renal tubules. A, Representative images of immunofluorescent staining after lentivirus infection. B, Western blot of Snail1 after puromycin selection. C, Quantification of protein expression by scanning densitometry in (B). D, The indicated protein levels were investigated in Snail1‐overexpression stable cells by western blot. E, Quantification of protein expression by scanning densitometry in (D). F, The cells were transfected with Snail1 siRNA and validated by western blot. G, Quantification of protein expression by scanning densitometry in (F). H, The cells were transfected with Snail1 siRNA and treated with TGF‐β1 and/or nodakenin; Next, the indicated protein levels were investigated. I, Quantification of protein expression by scanning densitometry in (H). Data represent the mean ± *SD* for at least three independent experiments. n.s. means no significant difference compared with the indicated group, *P < 0.05, ***P < 0.001 compared with the indicated group

To further elucidate whether nodakenin functions against renal fibrosis by suppressing the Snail1 expression, we overexpressed and silenced the Snail1 respectively. We first evaluated the fluorescence intensity of cells after transduction by converted microscopy. As shown in Figure 6A, the cells after lentivirus transduction induced a marked increase in fluorescence, suggesting successful transduction. Next, we measured the levels of Snail1 by western blot after puromycin treatment. The results indicated that Snail1 was obviously increased compared with the control group (details listed in Figure 6B,C). On the above basis, we determined the effect of nodakenin after TGF‐β1 treatment in Snail1 overexpression cells. In accordance with expectation, nodakenin failed to decrease the levels of fibronectin and α‐SMA in Snail1 overexpression cells (Figure 6D,E). Furthermore, Snail1‐silencing cell model was also established by siRNA transfection and detected by western blot (Figure 6F,G). However, addition of nodakenin did not alter the expression levels of fibronectin and α‐SMA in TGF‐β1 treated Snail1‐silencing cells (Figure 6H,I). In total, these observations indicated that nodakenin may at least partially function through block Snail1 expression.

## 4 DISCUSSION

Nodakenin, a small‐molecule coumarin isolated from the roots of *Angelicae gigas,* has been reported to posess multiple biological activities such as alleviating inflammation, enhancing cognitive function and so on.^12^, ^13^ However, whether nodakenin possesses the anti‐fibrotic actions remains poorly unclear. In our present study, we have determined the effect of nodakenin on UUO‐induced renal fibrosis and TGF‐β1‐treated renal epithelial cells. Our results indicated that the administration of nodakenin largely inhibited renal inflammation by attenuating the infiltration and polarization of macrophages, the production of cytokines and blunting the phosphorylation of NF‐κB p65. Meanwhile, we also observed that nodakenin treatment could improve the histological injuries and excessive deposition of the extracellular matrix such as fibronectin and collagen I. Moreover, the subsequent results demonstrated that nodakenin treatment blocked the phosphorylated smad3 and the aberrant expression of Snail1. To further validate whether nodakenin function against fibrosis by inhibiting the Smad3/Snail1 signal pathway, we constructed Snail1 overexpression cells by lentivirus transduction and found that nodakenin failed to decrease the levels of fibrotic genes in Snail1 overexpression cells, suggesting that the inhibition of renal fibrosis by nodakenin may at least partially function through Snail1 signal pathway in the obstructed kidneys.

Inflammation is a priming factor in the initiation of renal injury plays dominant role in renal fibrosis.^21^, ^22^, ^24^, ^25^ During this process, monocytes/macrophages are recruited to the glomerulus and renal interstitium. The activated kidney immune cells accelerate the production of pro‐inflammatory cytokines and in turn, aggravate the renal injury. In the current study, our results suggested that nodakenin treatment could dramatically inhibit the expression of multiple pro‐inflammatory cytokines such as TNF‐α, IL‐1β, iNOS and, the infiltration and polarization of macrophages. Consistent with the previous studies, our results have also revealed that nodakenin could prevent NF‐κB activation,^14^ indicating that nodakenin decreased the expression of pro‐inflammatory cytokines by interfering with NF‐κB activation. In total, alleviating inflammation response is one of the mechanisms by which nodakenin blunts the renal fibrosis.

The transcription factor Snail1 is a crucial protein in EMT and is mediated by multiple pathways.^26^ Of note, NF‐κB can induce the expression and stabilize Snail1 protein. In addition, Snail1 can also be activated by Smad3/4 complex. After that, Snail1 provoked the progression of fibrogenesis. Given the previous observations, inhibition of Snail1 protein should be an effective way to inhibit fibrosis. In this study, we found that nodakenin impacted the phosphorylated smad3. Meanwhile, findings from our research indicated that nodakenin is a potent inhibitor of Snail1, which suggested that nodakenin relieve the fibrosis at least partly by the suppression of Smad3/Snail1.

In conclusion, this study has shown that nodakenin can significantly inhibit UUO‐induced renal fibrosis in mice and TGF‐β1‐treated renal epithelial cells by regulating NF‐κB and Smad3 induced Snail1 expression, subsequently improving the inflammation and fibrogenesis in the obstructive kidney. The present study lays the groundwork for future research into a natural therapeutic agent for the treatment of renal fibrosis.

## CONFLICT OF INTEREST

The authors declare that there are no conflicts of interest.

## AUTHOR CONTRIBUTIONS

**Jianchun Li**: Data curation (equal); Writing‐original draft (equal). **Lu Wang**: Data curation (equal); Writing‐original draft (equal). **Ruizhi Tan**: Project administration (equal). **Sha Zhao**: Project administration (equal). **Xia Zhong**: Data curation (equal). **Li Wang**: Supervision (equal); Writing‐original draft (equal).

## Data Availability

The datasets generated during and/or analysed during the current study are available from the corresponding author on reasonable request.
